# Laser Modification of Sinus Node Function in Dogs

**DOI:** 10.19102/icrm.2018.091108

**Published:** 2018-11-15

**Authors:** Laszlo Littmann

**Affiliations:** ^1^Department of Internal Medicine, Carolinas Medical Center, Charlotte, NC, USA

**Keywords:** Ablation, inappropriate sinus tachycardia, sinoatrial node

I and my colleagues read with great interest the article by Weber et al.,^1^ in which the authors presented the results of laser catheter modulation of the sinus node under experimental conditions and in a patient with inappropriate sinus tachycardia.^1^ In their study, continuous-wave endocardial neodymium–yttrium–aluminum garnet (Nd:YAG) laser applications were delivered to sites showing the largest and earliest atrial potentials in the vicinity of the sinoatrial node. In the canine experiments, weekly electrocardiograms performed for three months showed permanent reduced heart rates. In the clinical case, the patient remained asymptomatic and her heart rate stayed normal during a follow-up period of 4.9 years.

We would like to raise awareness about an experimental study we performed almost three decades ago, in which we too employed continuous-wave Nd:YAG laser irradiation for the modification of sinus node function in dogs.^2^ There were distinct differences, however, between the study by Weber et al.^1^ and our study.^2^ First, we employed an epicardial rather than endocardial approach. Second, in our study, the origin of the sinus impulse (ie, the O-point) was electrically mapped before and during isoproterenol infusion. Epicardial laser photocoagulation was applied to the O-point observed during isoproterenol infusion and stepwise to remapped O-points until a 30% ± 5% decrease in the heart rate was achieved. Long-term effects were assessed by Holter monitoring and by electropharmacological testing preoperatively and up to 10 weeks or six months postoperatively. We found a consistent long-term decrease in the average 24-hour heart rates, the heart rates during pharmacologic autonomic blockade, and the maximum heart rates on isoproterenol. Holter monitoring did not show excessive bradycardic episodes even following the administration of propranolol. Histologic study of the irradiated area showed replacement by inflammatory cells as well as fibrosis and cartilage formation with surrounding normal cells and pacemaker-like cells at the caudal end of the sinoatrial node.

Our approach utilized the unique functional anatomy of the sinus node region, showing both a subepicardial location as well as widespread distribution and rate differentiation of the pacemaker complex, with predictable shifts occurring in the sites of impulse formation with changes in the autonomic tone and with certain pharmacologic interventions.^3^ Clinical studies are needed to determine whether the endocardial and substrate-based method employed by Weber et al.^1^ or our epicardial approach of selective, graded ablation of sinus node areas responsible for fast heart rates only is more effective for the control of inappropriate sinus tachycardia.
